# Drift transport of helical spin coherence with tailored spin–orbit interactions

**DOI:** 10.1038/ncomms10722

**Published:** 2016-03-08

**Authors:** Y. Kunihashi, H. Sanada, H. Gotoh, K. Onomitsu, M. Kohda, J. Nitta, T. Sogawa

**Affiliations:** 1 NTT Basic Research Laboratories, NTT Corporation, 3-1 Morinosato-Wakamiya, Atsugi, Kanagawa 243-0198, Japan; 2 Department of Materials Science, Tohoku University, 6-6-02 Aramaki-Aza Aoba, Aoba-ku, Sendai 980-8579, Japan

## Abstract

Most future information processing techniques using electron spins in non-magnetic semiconductors will require both the manipulation and transfer of spins without their coherence being lost. The spin–orbit effective magnetic field induced by drifting electrons enables us to rotate the electron spins in the absence of an external magnetic field. However, the fluctuations in the effective magnetic field originating from the random scattering of electrons also cause undesirable spin decoherence, which limits the length scale of the spin transport. Here we demonstrate the drift transport of electron spins adjusted to a robust spin structure, namely a persistent spin helix. We find that the persistent spin helix enhances the spatial coherence of drifting spins, resulting in maximized spin decay length near the persistent spin helix condition. Within the enhanced distance of the spin transport, the transport path of electron spins can be modulated by employing time-varying in-plane voltages.

## Abstract (web summary)

Spin-orbit effects in non-magnetic semiconductors allow for the manipulation of electronic spins in the absence of an applied magnetic field. Here, the authors exploit a persistent spin helix state in single quantum wells to enhance the coherence length of electronic drift transport.

## Graphical abstract

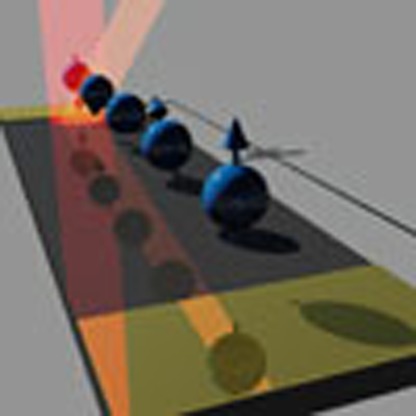

Transporting electron spins in spin–orbit coupled systems is the key to developing future spintronics devices and spin-based information processing^1^,^2^,^3^. Conduction electrons moving in semiconductors experience the spin–orbit interaction (SOI) as an effective magnetic field, which enables us to rotate the electron spins in the absence of an external magnetic field. At the same time, the SOI also has an adverse effect: the fluctuations in the effective magnetic field originating from the random scattering of electrons also cause undesirable spin decoherence^4^, which limits the length scale of spin transport. The enhancement of spin coherence during drift transport is essential for spin-based devices but has yet to be demonstrated.

The two-dimensional electron systems in semiconductors are promising for manipulating travelling spins with controllable SOIs. This is not just because of their high mobility, which leads to high-speed spin transfer, but also because of the ability to tune the strength of the Rashba SOI^5^ by applying a vertical electric field. Furthermore, the tunability of the SOI also provides the chance to balance the Dresselhaus and Rashba SOIs. This condition results in a robust spin structure for spin-independent scattering, namely a persistent spin helix (PSH), which has a helical spin density wave with an infinite spin lifetime. The PSH has been theoretically predicted^6^,^7^ and experimentally demonstrated by measuring the lifetime of the helical spin mode generated by the transient spin grating technique^8^.

Here we demonstrate the drift transport of electron spins in the PSH state, which has so far only been studied without lateral electric fields^8^,^9^,^10^. Recently, Yang *et al.*^11^,^12^ observed the drift transport of helical spin structures under unbalanced Dresselhaus and Rashba SOI conditions. We expect that the balanced condition realized by careful tuning of the gate-controlled Rashba SOI will effectively extend the length of the drift spin transport. In our experiment, a multi-cycle spin precession was observed for a specific direction, while it was completely quenched for other orthogonal directions, reflecting the achievement of an exactly balanced SOI condition. A theoretical model reproduced the spin distributions well and revealed that the spin decay length is maximized near the PSH condition. Within the enhanced distance of the spin transport, the transport path of electron spins can be modulated by employing time-varying in-plane voltages, suggesting the possibility of coherent spin manipulation via geometrically controlled SOIs.

## Results

### Sample design and measurement principle

We used two-dimensional electron systems in (001) III–V semiconductor quantum wells (QWs), which provide SOIs suited to the direct observation of a PSH. The *k*-linear term of the SOI Hamiltonian for this system is written as *H*_SO_=(*α*−*β*)*k*_*y*_*σ*_*x*_−(*α*+*β*)*k*_*x*_*σ*_*y*_, using a coordinate system with base vectors 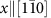, 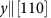 and 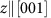. The parameters *α* and *β* represent the strengths of Rashba and Dresselhaus SOIs, respectively. In the balanced condition where *α*=*β* (*α*=−*β*), the effective magnetic fields become unidirectional along *y*(*x*) and proportional to *k*_*x*_(*k*_*y*_), respectively. This results in the appearance of a helical spin mode or PSH that is robust against spin-independent scattering. An optical technique using the magneto-optic effect enables us to access the helical mode through the distribution of the *z*-component of the spins, which forms stripe patterns in a two-dimensional plane^9^,^10^.

**Figure 1:**
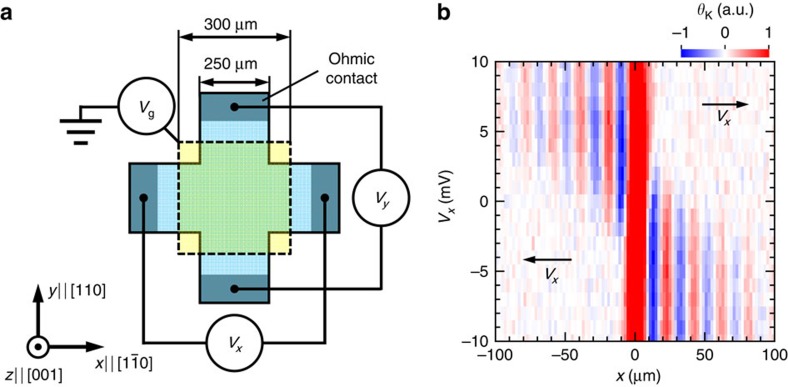
Creation of drift spin flows in a cross-shaped device. (**a**) Schematic of top view of our samples. (**b**) In-plane electric field dependence of spin distribution in steady state in 25-nm-thick QW at *V*_g_=−4.45 V.

To investigate the drift transport of helical spin states, we designed two samples, each of which contains a single GaAs/AlGaAs QW (*d*_QW_ thicknesses of 15 and 25 nm) embedded in a high electron mobility transistor structure. The energy band structures calculated by the *k*·*p* perturbation theory^13^ allowed us to estimate the strengths of *α* and *β*, which are close to the PSH condition (*α*∼*β*) for the 25-nm-thick QW, whereas they are far from the PSH condition (*α*<*β*) for the 15-nm-thick QW. The samples were grown by molecular-beam epitaxy and fabricated into cross-shaped mesa structures with four ohmic contacts (Fig. 1a). The samples were covered with semi-transparent Au gate electrodes. The voltage applied to the ohmic contacts (*V*_*x*_ or *V*_*y*_) creates electric fields to transport electrons in the QW plane, while the top gate voltage (*V*_g_) allows us to tune the Rashba SOI strength simultaneously^5^.

We employed Kerr microscopy to measure the spin dynamics of drifting electrons. A circularly polarized pump light from a continuous wave (cw) Ti:sapphire laser generates electron spins oriented in the *z* direction at a certain position defined as (*x*, *y*)=(0, 0) on the QW, and a linearly polarized light probes the magneto-optic Kerr rotation *θ*_K_, which is proportional to the spin density at the focused position. For the microscopic measurement of spin coherence, the pump and probe lights were focused on the sample with 6 and 3 μm diameter spots, which are smaller than the typical spin precession length resulting from an SOI (∼20 μm) estimated for the present QWs.

### Kerr imaging of drift spin transport

The application of an in-plane electric field changes the spin transport regime significantly. Figure 1b shows a one-dimensional scan of *θ*_K_ along the *x*-axis measured for different in-plane voltages *V*_*x*_ and a fixed *V*_g_ of −4.45 V applied to the 25-nm-thick QW. The symmetrical diffusion of the spins at *V*_*x*_=0 V shows the spin precession caused by the SOI, as reported previously^9^, but it only appears in a small area |*x*|≲10 μm. When we increase |*V*_*x*_|, the signal becomes asymmetric, indicating that spins flow in the electron drift direction. The Kerr signals observed under in-plane electric fields provided real-space imaging of the drifting spins in a steady state, whose dynamics have been previously investigated in QWs only with a Dresselhaus SOI by spin grating spectroscopy^11^,^12^. The oscillating signal of drifting spins is maintained for a much longer distance than at *V*_*x*_=0 V, thus enabling us to determine the SOI strengths more precisely.

**Figure 2:**
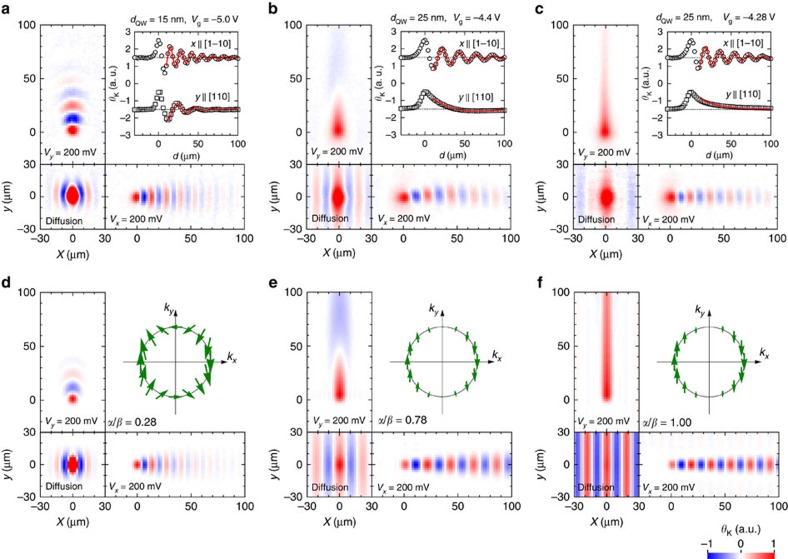
Mapping of spins with/without in-plane electric fields. (**a**–**c**) Kerr rotation image of spins with/without in-plane electric fields in 15-nm-thick QW at *V*_g_=−5.0 V (**a**), and 25-nm-thick QW at *V*_g_=−4.4 V (**b**) and −4.28 V (**c**). Insets are cross-sectional patterns of drifting spins in the *x* and *y* directions. (**d**–**f**) Spatial mapping of drifting spins simulated by SDD model, corresponding to **a**–**f**. Insets show effective magnetic fields in *k*-space for given SOI parameters.

The effects induced by the coexistence of the Rashba and Dresselhaus SOIs appear as anisotropy of the spatial precession frequency for different moving directions. Figure 2a–c maps the spatial distribution of electron spins drifting in the 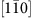 and [110] directions as well as that of spins without drift motion. In the 15-nm-thick QW under *V*_g_=−5.0 V (Fig. 2a), the precessions have different spatial frequencies for the *x* and *y* directions. This is attributed to the anisotropic effective magnetic fields, which are expected to be proportional to |*α*+*β*| for *k*_*x*_ and |*α*−*β*| for *k*_*y*_. Because of the moderate anisotropy of the effective magnetic field in the 15-nm-thick QW, the *θ*_K_ oscillations caused by the spin precession have several periods in both directions. On the other hand, in the 25-nm-thick QW (Fig. 2b,c) the extreme anisotropy has a large effect on the spatial precession frequency, namely the precessions of spins drifting along the *y*-axis slow almost to a halt. Such a slow precession cannot be resolved by the stripe patterns in the small region observed in the regime where no in-plane electric field is applied^9^. Instead, the extended transport length for drifting spins along *y* reveals a rotation of more than *π* caused by a small effective magnetic field. By carefully tuning *V*_g_ from −4.40 V (Fig. 2b) to −4.28 V, (Fig. 2c), we achieved a condition where the spin precession was halted completely even in the drifting regime. This indicates the uniaxial configuration of the effective magnetic fields, which provide the exact SU(2) symmetry with great accuracy.

For a quantitative analysis of the spin dynamics during drift transport, we extracted two parameters that characterize the two-dimensional distribution of the Kerr rotation signal. We fitted the cross-sectional profile of the data with a function *θ*_K_(*d*)=exp(−*d*/*l*_s_) cos(2*πd*/*L*_SO_), where *d* is the distance from the origin, *l*_s_ is the spin decay length caused by the spin diffusion and/or decoherence, and *L*_SO_ is the spin precession length in which electron spins rotate a full cycle. As shown in Fig. 2a–c, the data are fitted well and allow us to obtain the SOI parameters by using the following relations:


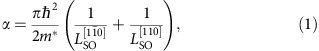



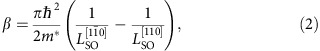


where *ℏ* and m* are Planck's constant and the effective mass of an electron, respectively. The *L*_SO_ obtained by the fitting and SOI parameters calculated from Equations (1) and (2) are summarized in Table 1. We confirmed that *α* is tuned by the gate voltage and has the same value as *β* at *V*_g_=−4.28 V. We also found that there was a slight variation in *β*=*γ*<*k*_*z*_^2^>, where *γ* is a material constant, when modifying the gate voltage, which is due to the gate voltage dependence of the wavenumber <*k*_*z*_^2^> quantized in the *z*-direction.

### Theoretical simulation with spin drift-diffusion model

The spatial evolution of spins observed in Fig. 2a–c shows excellent agreement with a theoretical calculation (Fig. 2d–f) performed using a spin drift-diffusion (SDD) equation:


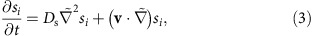


where *s*_*i*_ is the *i*-th component of the spin vector, *D*_s_ is the spin diffusion coefficient, **v** is the drift velocity, and 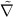 is the covariant derivative in a system treated with a spin-dependent SU(2) vector potential^14^. We transformed Equation (3) to the Fourier space and obtained the equation:


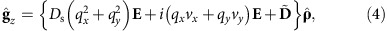


where *q*_*i*_ is the wavenumber of the spin mode in the *x* and *y* directions and **E** is an identical matrix. 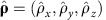 and 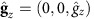 are the spin polarization and generation matrix in the Fourier space, respectively. We used a two-dimensional Gauss function with a 6 μm half width at half maximum for the steady generation of spin polarization. The first and second terms in Equation (4) represent the spin diffusion and drift transport of spins, respectively, whereas 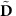 is a matrix including the spin rotation and relaxation given by,


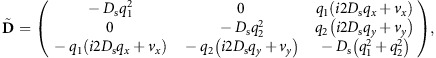


where *q*_1_=2m*(*α*+*β*)/*ħ*^2^ and *q*_2_=2m*(*α*−*β*)/*ħ*^2^. The spin distribution in real space can be obtained from the inverse Fourier transform of the solution of Equation (4). The contribution of the cubic Dresselhaus term is neglected in the simulation because the Fermi wavenumber *k*_F_ and drift wavenumber *k*_d_=m*v_d_/*ħ* are estimated to be 10^7^ m^−1^ or smaller for the condition used in the experiment, thus the cubic term (Ω_cubic_=*γk*^3^/*ħ*) should be one or two orders smaller than the linear term (Ω_linear_=2*βk*/*ħ*). The *D*_s_ values were estimated from the lateral broadening of the Kerr rotation signal for the spins drifting in the *x* direction (shown in Table 1), and the **v** values were obtained from a time-resolved measurement of the spin transport in the same samples. The calculated results shown in Fig. 2d–f well reproduce all the spin dynamics behaviours including the precession, expansion, and decay of the drifting and diffusing spins.

**Figure 3:**
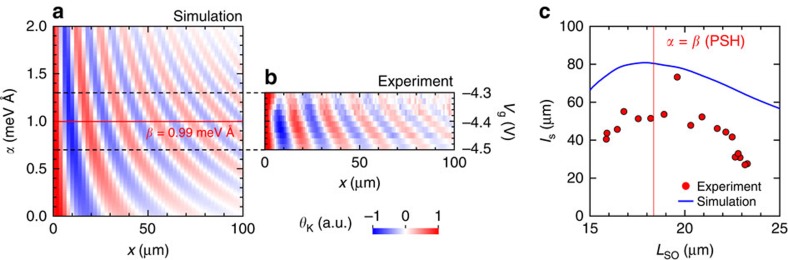
SOI dependence of spin dynamics of drifting spins. (**a**) Comparison of simulated and experimental spin distribution driven by *V*_*x*_=50 mV in the *x* direction. In the simulation, we directly varied the Rashba SOI parameters *α* and the spin distribution is shown as a function of *α*, whereas (**b**) the experimental data are plotted as a function of gate voltage, which modulates the Rashba SOI. Note that the gate voltage shown in this figure does not correspond to that in other experiments because the measurements were carried out on different days leading to different offset voltages. (**c**) Spin decay length as a function of spin precession length.

Our experimental and calculated results show that the PSH condition is favourable for long-distance drift spin transport, which agrees with the prediction by random walk theory^15^. Figure 3a shows the result of an SDD simulation, in which the *s*_*z*_ values are plotted as a function of *x* and *α* for the drift spins moving in the *x* direction. Because we fixed the *β* value in the calculation, we can expect the spatial precession frequency 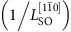 to increase linearly with *α*, according to Equations (1) and (2). Similar behaviour was observed experimentally for drifting spins around the PSH condition via the gate control of the Rashba SOI (Fig. 3b). Assuming that the precession period in Fig. 3b depends only on *α*, we can compare the effect of *α* on the transport length by plotting *l*_s_ versus *L*_SO_, where both parameters can be extracted by the fitting procedure used in Fig. 2. The obtained *l*_s_ values are plotted as a function of *L*_SO_ in Fig. 3c. In both the experiment and the simulation, *l*_s_ decreases as *L*_SO_ diverges from the balanced condition *α*=*β*, indicating that the suppression of the D'yakonov- Perel' (DP) spin relaxation caused by PSH also appears in the spin transport length. It should be noted that the fact that the spin decay length in the experiment is shorter than in the simulation could be due to spin decoherence caused by the cubic Dresselhaus term although its magnitude is one order smaller than the linear Dresselhaus term.

### Modulation of spin transport path by time-varying electric fields

**Figure 4:**
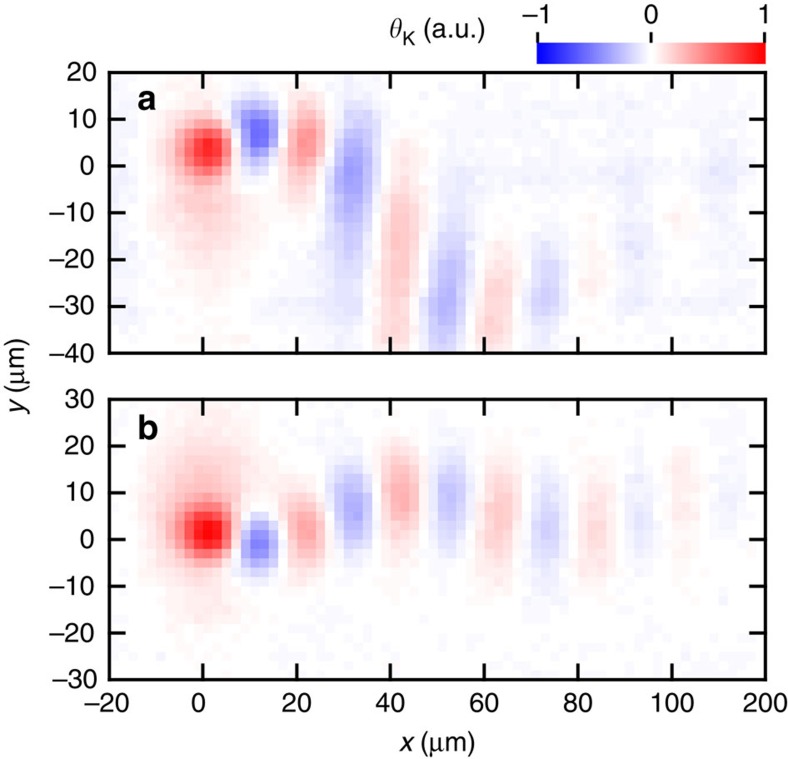
Dynamic modulation of transport path of drifting spins. (**a**,**b**) Drift spin transport driven by 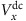
=100 mV with sinusoidal voltage 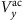
=200 mV applied in the *x* and *y* direction, respectively. The frequency of the 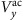 was adjusted to the repetition frequency of the mode-locked laser, 82.46 MHz (**a**) or twice the frequency (**b**).

We can transport the helical spin state along arbitrary trajectories by using a time-dependent in-plane electric field. We applied a sinusoidal ac voltage 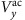 with frequency *f* in the *y* direction and dc voltages 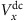 in the *x* direction. To excite spins at the timing of a certain phase of 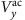, the initial spins were excited with a pulsed pump light from a mode-locked laser whose repetition frequency was synchronized with *f* or *f*/2, whereas the Kerr signal was detected with a probe light from a cw laser. When a sinusoidal voltage was applied in the transverse direction of the drift spin transport, a clear spin flow along a winding path was observed (Fig. 4). The stripe patterns in the spin packet are maintained regardless of transport trajectories. This is because, in a PSH condition, spins always precess in an *x*–*z* plane and the precession phase depends only on *x*. For all the transport paths, the spin phase was conserved even at distances much longer than the precession periods, and thus the technique will be beneficial for further exploitation of the SOI in drift spin manipulation.

The results presented here show that spin coherence with a balanced SOI can be efficiently transferred to a distant place without the aid of any static narrow quasi-one-dimensional channels. Because we can use lateral electric fields for high-mobility electron systems, the transport speed can be much faster than that in bulk systems^16^,^17^ or acoustic waves^18^,^19^,^20^, which have been used in previous spin-transport experiments. If the balanced condition is sufficiently maintained, we may also manipulate the spin states by using the gate bias voltage or trajectory-controlled quantum operation^19^,^21^. Further development of this technique will advance the field of semiconductor spintronics including the physics of a spin-charge coupled system^22^,^23^ as well as applications for future spin devices^6^,^24^,^25^.

## Methods

### Sample fabrication

The samples were 15- and 25-nm-thick modulation-doped Al_0.3_Ga_0.7_As/GaAs/Al_0.3_Ga_0.7_As single QWs grown by molecular-beam epitaxy on a (001) semi-insulating GaAs substrate. The QW was located 120 nm below the surface, whereas three *δ*-doping layers were embedded 50, 100 and 105 nm below the surface. The epitaxial wafers were fabricated into cross-shaped structures with four ohmic contacts consisting of AuGe (260 nm) and Ni (40 nm) layers. The central part of the structure was covered with a 7-nm-thick Au gate electrode through which we could obtain the optical signal from the QW.

### Optical measurement

We employed spatially resolved Kerr rotation microscopy in which spin polarization along the *z*||[001] direction was generated with a circularly polarized pump light (100∼275 μW average power) from a cw Ti:sapphire laser and detected with a linearly polarized probe light (1.0∼6.8 μW). To excite spins at the timing of a certain phase of 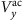, we undertook an experiment involving drifting spin path modulation induced by time-dependent in-plane electric fields. The initial spins were excited with a pulsed pump light from a mode-locked laser whose repetition frequency was synchronized with the frequency *f* of the in-plane electric field, whereas the Kerr signal was detected with a probe light from a cw laser. The wavelengths of both the pump and probe lights were adjusted to maximize the Kerr signals for each QW and gate voltage. The polarization of the pump light was modulated between left- and right-circular polarizations at 50.1 kHz, and the probe light was chopped with an acousto-optic modulator at 52.0 kHz. The difference frequency (1.9 kHz) was used as a reference for lock-in detection. The full width at half maximum spot size of the normally incident probe beam was ∼3 μm, whereas the waist size of the obliquely incident pump beam was 6 μm and its spot on the sample was slightly elongated in the 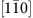 direction. The position of the probe light spot was scanned in the QW plane for spatially resolved Kerr rotation measurements. All the measurements were carried out at 8 K.

## Additional information

**How to cite this article:** Kunihashi, Y. *et al.* Drift transport of helical spin coherence with tailored spin–orbit interactions. *Nat. Commun.* 7:10722 doi: 10.1038/ncomms10722 (2016).

## Figures and Tables

**Table 1:** Spin-related parameters obtained from the experiments.

***d*_QW_ (nm)**	**15**	**25**	**25**
***V*_g_ (V)**	**−5.0**	**−4.4**	**−4.28**
*D*_*s*_ (cm^2^ s^−1^)	60.8±10.1	40.8±9.2	17.6±8.7
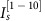 (μm)	29.6±4.0	37.8±6.6	37.5±5.9
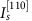 (μm)	21.2±3.4	35.0±4.2	23.2±5.2
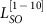 (μm)	13.5±0.2	21.5±0.7	18.3±0.4
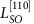 (μm)	23.7±1.5	172.9±15.7	—
*α* (meV Å)	0.58±0.04	0.74±0.07	0.99±0.02
*β* (meV Å)	2.10±0.13	0.95±0.09	0.99±0.02

The parameters (*D*_s_, *l*_s_, *L*_SO_, *α* and *β*) and their uncertainties (s.d.) for different *d*_QW_ and *V*_g_ values are derived by a least-squares fit.
